# Supplementary material for: Inaccessible LCG Promoters Act as Safeguards to Restrict T Cell Development to Appropriate Notch Signaling Environments
Source: Stem Cell Reports. 2021 Mar 25;16(4):717–26. doi: 10.1016/j.stemcr.2021.02.017 (PMC8072033; doi:10.1016/j.stemcr.2021.02.017)
Supplement: Document S1. Figures S1–S3 [file mmc1.pdf]

Stem Cell Reports, Volume *16*

Supplemental Information

Inaccessible LCG Promoters Act as Safeguards to Restrict T Cell Development to Appropriate Notch Signaling Environments

Suzanne Furuyama, Qian "Vicky" Wu, Barbara Varnum-Finney, Richard Sandstrom, Wouter Meuleman, John A. Stamatoyannopoulos, and Irwin D. Bernstein

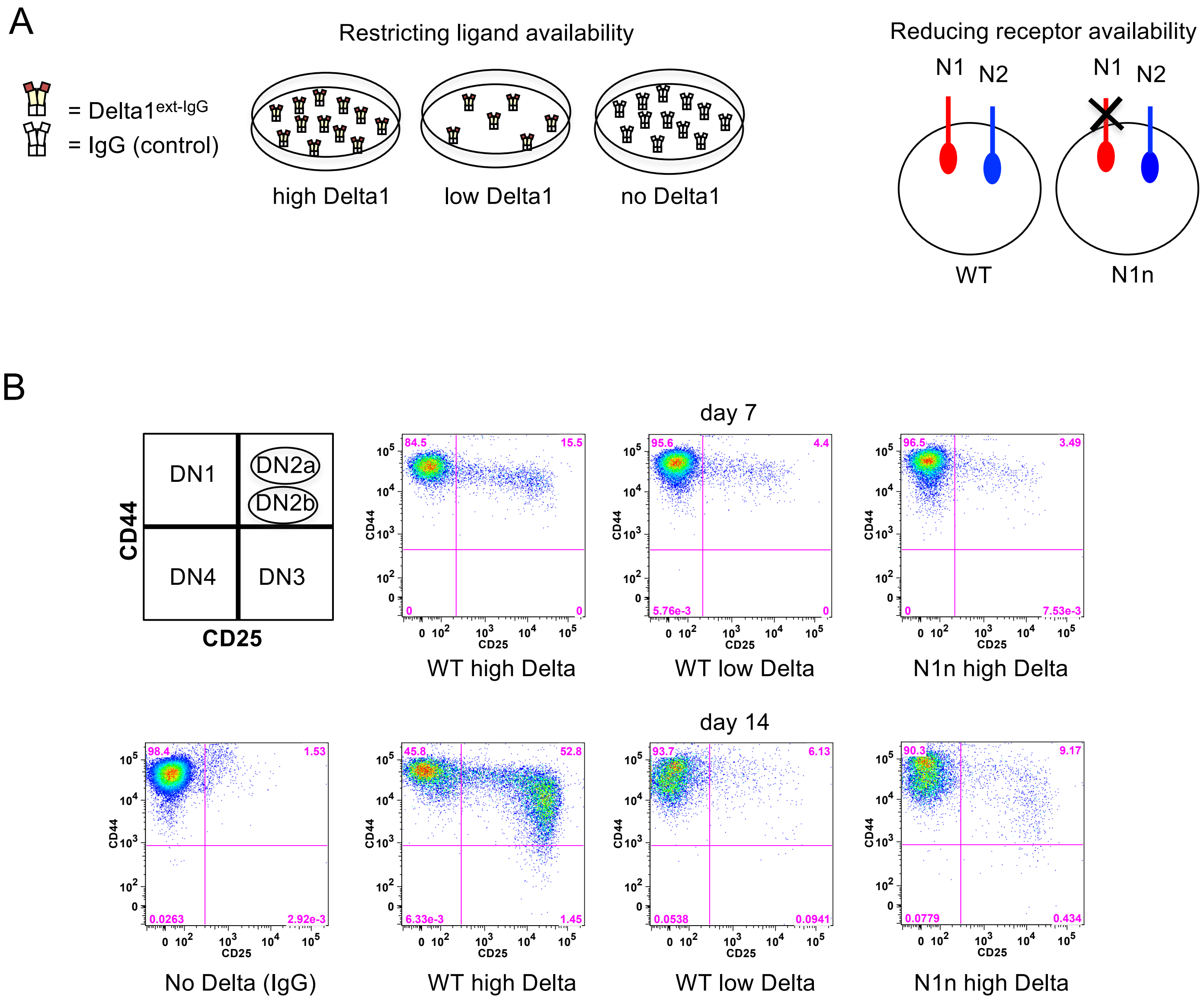

Figure S1: Use of an immobilized Notch ligand culture system to control the level of Notch activation, and ultimately cell fate outcome, Related to Figure 1. A. Schematic representation of the ex vivo culture system used. To vary the amount of ligand presented during ex vivo culture, flasks are coated with low (0.75µg/ml) or high (5µg/ml) concentration of Delta1$^{ext\text{-}IgG}$. To reduce the amount of available Notch receptor, we utilized a mouse strain bearing a Cre-mediated conditional *Notch1* deletion (Radtke et al., 1999). **B.** Representative dot plots showing the percent of CD25^{+}CD44^{+} Double Negative 2 (DN2) precursors generated following 7 and 14 days of LSK culture on different doses of Delta1$^{ext\text{-}IgG}$ or no Delta (IgG) control. Numbers within dot plots denote the percentage of the population within the respective quadrants.

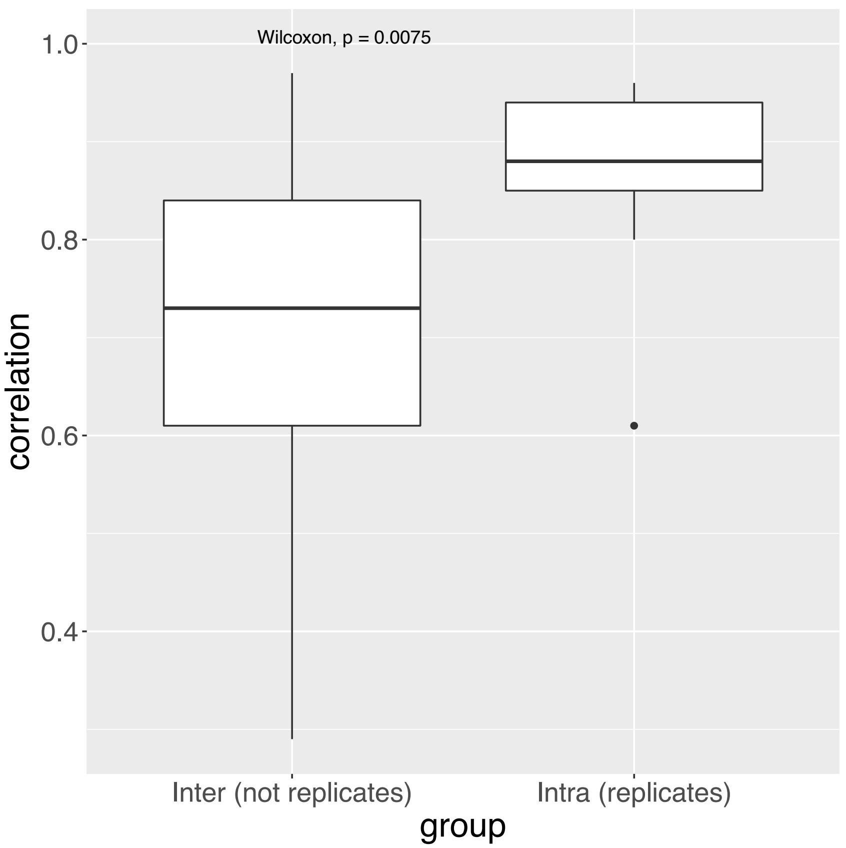

Figure S2: Assessment of DNase-seq replicate concordance, Related to Figures 1 & 2. Boxplot describing the correlation between datasets from the same developmental stage (Intra) as well as among different developmental stages (Inter). The median correlation coefficient, R, among intra-stage replicates is 0.88, while that of inter-stage non-replicates is 0.73. The non-parametric Wilcoxon rank test was used to assess statistical significance among groups, p = 0.0075.

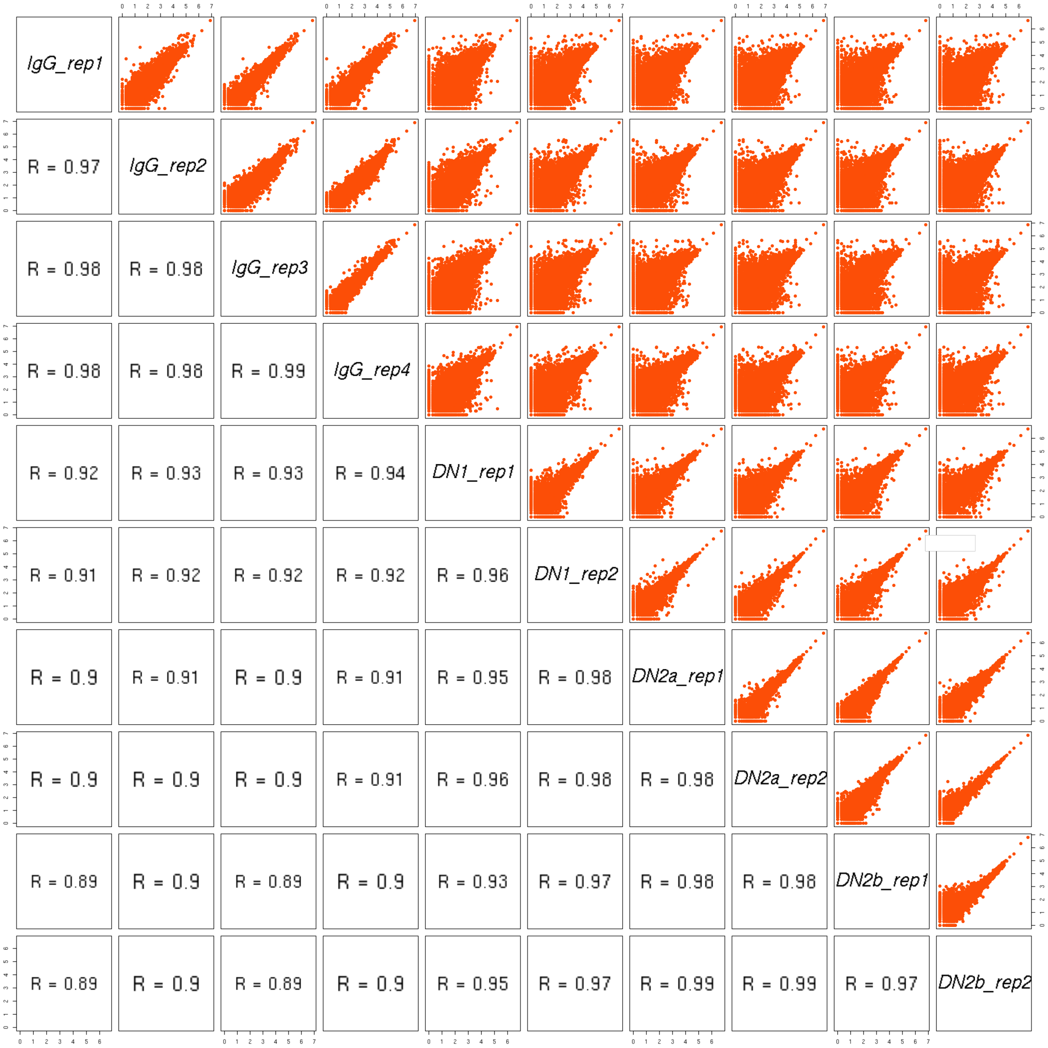

Figure S3: Assessment of RNA-seq replicate concordance, Related to Figure 2. Scatterplots showing the correlation between each dataset, both the same (biological replicates) and different (non-biological replicates) developmental stages. The X and Y-axes represent $\log_{10}$(gene expression count +1). The correlation coefficient, R, is also noted.

Supplemental Table Legends:

Table S1: DNase-seq & RNA-seq Dosage Screen Results, Related to Figure 2.

Sheet 1: ***Master Table***. Includes all DNase-seq and RNA-seq results used to identify high dose Notch dependent and low dose Notch responsive promoters.
Sheets 2-4: '***Unique***' sheets represent a simplified list of unique gene promoters and their coordinates that overlap with a DHS in each dosage class (high dose, low dose part1, low dose part2).

Table S2: Gene Ontology and CpG analysis, Related to Figures 2 & 4.

Sheet 1: Reference gene list for Gene Ontology Analysis (GOrilla).
Sheet 2: GOrilla output for combined low dose Notch responsive genes.
Sheet 3: GOrilla output for high dose Notch dependent genes.
Sheet 4**:** Observed/expected CpG ratio among the promoters of Notch dose responsive subgroups
