# Supplementary material for: Inaccessible LCG Promoters Act as Safeguards to Restrict T Cell Development to Appropriate Notch Signaling Environments
Source: Stem Cell Reports. 2021 Mar 25;16(4):717–26. doi: 10.1016/j.stemcr.2021.02.017 (PMC8072033; doi:10.1016/j.stemcr.2021.02.017)
Supplement: Document S2. Article plus Supplemental information [file mmc4.pdf]

Stem Cell Reports
Article

OPEN ACCESS

# Inaccessible LCG Promoters Act as Safeguards to Restrict T Cell Development to Appropriate Notch Signaling Environments

Suzanne Furuyama,[1] Qian "Vicky" Wu,[1] Barbara Varnum-Finney,[1] Richard Sandstrom,[3,4] Wouter Meuleman,[3] John A. Stamatoyannopoulos,[3,4,5] and Irwin D. Bernstein[1,2,*]
[1]Clinical Research Division, Fred Hutchinson Cancer Research Center, Seattle, WA 98109, USA
[2]Department of Pediatrics, Division of Pediatric Hematology/Oncology, University of Washington, Seattle, WA 98195, USA
[3]Altius Institute for Biomedical Sciences, Seattle, WA 98121, USA
[4]Department of Genome Sciences, University of Washington, Seattle, WA 98195, USA
[5]Department of Medicine, Division of Oncology, University of Washington, Seattle, WA 98195, USA
*Correspondence: ibernste@fredhutch.org
https://doi.org/10.1016/j.stemcr.2021.02.017

## SUMMARY

T cell development is restricted to the thymus and is dependent on high levels of Notch signaling induced within the thymic microenvironment. To understand Notch function in thymic restriction, we investigated the basis for target gene selectivity in response to quantitative differences in Notch signal strength, focusing on the chromatin architecture of genes essential for T cell differentiation. We find that high Notch signal strength is required to activate promoters of known targets essential for T cell commitment, including *Il2ra*, *Cd3ε*, and *Rag1*, which feature low CpG content (LCG) and DNA inaccessibility in hematopoietic stem progenitor cells. Our findings suggest that promoter DNA inaccessibility at LCG T lineage genes provides robust protection against stochastic activation in inappropriate Notch signaling contexts, limiting T cell development to the thymus.

## INTRODUCTION

In numerous developing systems, Notch modulates the decisions that determine the fate of stem cells and their progeny (Artavanis-Tsakonas et al., 1999; Lai, 2004). Notch is a transmembrane receptor that is activated by binding of ligand to its extracellular domain. Upon ligand binding, the Notch receptor is proteolytically cleaved, releasing its intracellular domain (ICD). The Notch ICD translocates to the nucleus and functions as a transcriptional activator, directed to target genes by association with the RBPJ$_\kappa$ DNA-binding protein (for a review see Kopan and Ilagan, 2009). In mammals, there are four Notch receptors (NOTCH1 to NOTCH4) and five canonical Notch ligands (DLL1, DLL3, DLL4 and JAG1, JAG2).

With respect to the T lineage, T cell differentiation is limited to the Notch ligand-rich thymic microenvironment (Zuniga-Pflucker, 2004), where *Notch1* has been shown to be essential for T cell development (Radtke et al., 1999). *In vivo* swapping of the ICDs of *Notch1* and *Notch2* paralogs has shown that either ICD is capable of promoting T cell development (Liu et al., 2015), suggesting that thymic restriction is dependent on the high Notch signal strength resulting from activation of the elevated levels of NOTCH1 found in the thymus. This suggestion is supported by studies showing that *ex vivo* cultivation of enriched hematopoietic stem progenitor cells (HSPCs) with immobilized Notch ligands (Varnum-Finney et al., 2000) promotes T cell differentiation only at high density (Dallas et al., 2005; Delaney et al., 2005), and partial reduction of *Notch1* expression *in vivo* impairs alpha-beta T cell development (Washburn et al., 1997). While the basis by which quantitative differences in Notch signaling are interpreted at target genes has not been elucidated, the selective use of NOTCH1 to induce an activation signal sufficient to promote the development of T cells suggests that Notch-responsive gene promoters are not equivalent in their transcriptional competence.

The notion of differential transcriptional competence has been established by the finding that the majority of gene promoters, including housekeeping genes, display histone markings associated with an active or "poised" chromatin state, while the promoters of many lineage-associated genes lack histone modifications associated with active, suppressed, or "poised" states (Mikkelsen et al., 2007), suggesting they are in a default closed chromatin confirmation. Here, we examined the relationship between DNA accessibility and the dose-dependent transcriptional response of Notch target genes essential for early T cell development. Our data suggest that high-Notch dose-dependent promoters, including *Il2ra*, *Cd3ε*, and *Rag1*, feature LCG promoters and DNA inaccessibility in the ground HSPC state, acquiring promoter DNA accessibility only upon exposure to high levels of Notch signaling. These data implicate chromatin conformation and promoter CpG content as critical features in assuring appropriate Notch-mediated cell-fate outcome. These findings further suggest that the closed chromatin conformation of LCG genes may have evolved to act as signal safeguard, preventing stochastic lineage commitment in inappropriate signaling contexts.


This is an open access article under the CC BY license (http://creativecommons.org/licenses/by/4.0/).

## RESULTS

### *Ex Vivo* System to Study Notch Dose-Dependent Promotion of Early T Cell Precursors

Notch signaling contributes to the early phases of T cell development, the first being the generation of $CD4^-$, $CD8^-$ double-negative 1 (DN1) ($KIT^{++}CD44^+CD25^-$) cells (Chen et al., 2019). These cells progress through the DN2a ($KIT^{++}CD44^+CD25^+$) stage prior to committing to the T lineage pathway with the first productive T cell receptor rearrangements, marked by the appearance of the DN2b ($KIT^+CD44^{lo}CD25^+$) subpopulation (Yui and Rothenberg, 2014). DN2 cells are essentially absent upon conditional deletion of *Notch1 in vivo* (Radtke et al., 1999), a condition preventing maximal activation of Notch signaling. *Ex vivo*, the developmental progression through the DN2 stages of early T cell development can be recapitulated by culturing freshly isolated murine HSPCs ($Lin^-SCA1^+KIT^+$ [LSK] cells) on a high density of immobilized Notch ligand (Dallas et al., 2005; Varnum-Finney et al., 2011) (Figure S1). Using this approach, we sought to identify Notch dose-dependent changes in DNA accessibility that accompany an HSPC as it progresses through the DN stages of T cell development.

### Use of Differential DNA Accessibility to Identify Notch Dose-Responsive Promoters in Early T Cell Development

Treatment of chromatin with the DNA endonuclease DNase I has been used to map accessible or "open" regulatory regions, referred to as DNase I hypersensitive sites (DHSs) (Stalder et al., 1980; Wu et al., 1979a, 1979b). We applied DNase I digestion followed by deep sequencing (DNase-seq) to identify genome-wide accessible regions (John et al., 2013) in the nuclei of biologically distinct LSK cells freshly isolated from murine marrow as well as stage-specific DN cells isolated from *ex vivo* LSK culture on high-density immobilized Notch ligand or no ligand control (no Notch) following T cell induction (Gene Expression Omnibus series accession GEO: GSE142739).

Using an unbiased approach (hotspot2), we identified 170,269 DHS regions genome wide, across all stages of T cell development (Figures 1A and S2). Of these, more than 126,000 (~74.4%) have been previously observed in a genome-wide atlas of mouse DHSs (Vierstra et al., 2014). Of the ~170,000+ DHSs, the midpoint of 27,000+ (~16%) was found to directly overlap with the promoters (±1 kb from transcription start site) of protein-coding genes. While there is currently no gold-standard database of mouse enhancers, we also assessed the fraction overlapping with enhancers using the catalog of candidate *cis*-regulatory elements (cCREs) derived from the ENCODE Project Consortium (2020). In total, ~46% of our ~170,000 identified DHSs overlap with promoters or proximal/distal enhancer elements.

As shown in Figure 1B, approximately 26,000 DHSs were shared across all stages of development. Of the remainder, a large proportion (34,000+) was shared with a previous developmental stage, while smaller subsets were acquired *de novo*, in a stage-specific manner. These three accessibility classes are consistent with those observed during the differentiation of embryonic stem cells along the cardiac lineage (Stergachis et al., 2013).

Promoter-associated DHSs were screened for Notch responsiveness by assessing differential promoter accessibility between biological replicates at each developmental stage (e.g., LSK versus DN1, DN1 versus DN2b, and so forth) and cells grown in the absence of Notch ligand using the non-parametric method ChIPtest (Wu, 2013; Wu et al., 2015). Rather than modeling total read counts in a given window such as DESeq (Anders and Huber, 2010) or calling peaks such as MACS (Zhang et al., 2008), ChIPtest models the spatial profiles of read counts in each DHS region and uses a non-parametric test to develop a differential accessibility score. Bonferroni adjustment was applied to correct for multiple testing, and the DNA accessibility threshold is based on an adjusted p value of <0.05.

Notch-responsive promoters were further subdivided into dose-response subgroups by applying differential accessibility criteria (Figure 1C) developed from observed correlations between Notch dosage and developmental state (Figure S1B). As low-dose Notch signaling fails to allow cells to progress beyond the DN1 stage of development, we reasoned that low-dose Notch-responsive promoters would fall into two categories: (1) those whose promoter accessibility is acquired *de novo* following the transition of LSK cells to DN1 cells (low-dose-responsive I) or (2) those whose promoter is accessible in LSK cells but whose extent of accessibility is increased in DN1 cells (low-dose-responsive II). Conversely, since high-dose Notch signaling is required for progression to the DN2 stage, we reasoned that high-dose-dependent promoters would be inaccessible in LSK/DN1 cells and acquire DNA accessibility *de novo* in DN2 cells, specifically DN2b cells.

To confirm whether the observed DNA accessibility patterns are relevant to lineage determination, we correlated the developmental timing of Notch dose-dependent changes in promoter DNA accessibility with the onset of gene expression. To do so, we assessed genome-wide changes in mRNA expression of stage-specific DN cells isolated from *ex vivo* cultured LSK cells using RNA sequencing (RNA-seq) (Figure S3). Read counts for each DN stage or "no Notch" control samples (series accession GEO: GSE142739) were used in pairwise comparisons to calculate $\log_2$ fold-change ($\log_2$FC) values. As described in Figure 1C, low-dose genes displaying $\log_2FC \geq 2$ between DN1 and control or high-dose genes

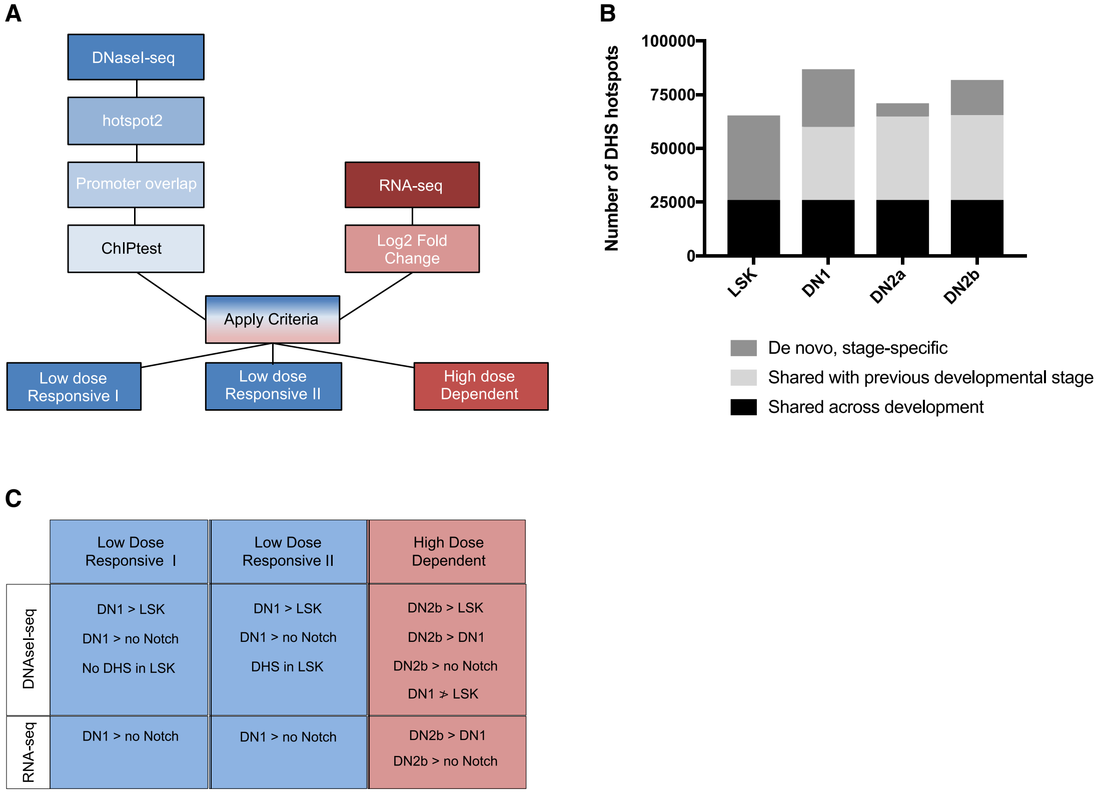


**Figure 1. Unbiased Genome-Wide Approach to Identify Notch Dose-Dependent Changes in Promoter DNA Accessibility and Gene Expression Accompanying Early T Cell Development**

(A) Flow chart illustrating the steps of our *de novo* discovery approach used to identify Notch dose-responsive genes.

(B) Graph representing the number of DHSs identified in LSK, DN1, DN2a, and DN2b stages of development. At each developmental stage, DHSs are further classified based on whether the DHS is shared across all stages of development, shared with the previous developmental stage, or acquired *de novo*.

(C) Criteria used to identify low-Notch dose-responsive and high-Notch dose-dependent promoters.

See also Figures S1 and S2.

displaying $\log_2 FC \geq 2$ between DN2b and control as well as DN2b and DN1 were selected. Following the implementation of both DNA accessibility and gene expression criteria, we identified 114 unique low-dose-responsive and 38 unique high-dose-dependent promoters (Figure 2A and Table S1). Scatterplots showing the correlation between the DNA accessibility score and gene expression for genes within each criteria class are shown in Figure 2B.

We performed gene ontology (GO) enrichment analysis on our lists of low-dose-responsive and high-dose-dependent genes using GOrilla (Eden et al., 2007, 2009). No biological process GO term was significantly enriched among the low-dose-responsive subset (for full GOrilla output, see Table S2). In contrast, among the high-dose-dependent genes, T cell-associated GO terms predominate, with T cell differentiation (false discovery rate [FDR] q value = $2.32 \times 10^{-4}$) being the most significant. Importantly, identified high-dose-dependent genes include Notch targets essential for progression through T cell commitment, such as *Il2ra* (Maillard et al., 2006), a subunit of the interleukin-2 (IL-2) receptor and a marker of progression through the DN stages of T cell development; *Cd3ε* (Clevers et al., 1989; De Smedt et al., 2007), a member of the T cell receptor complex; *Rag1* (McBlane et al., 1995), a gene required for T cell receptor recombination; and *Bcl11b* (Li et al., 2010), the T lineage commitment factor. Tracks showing the pattern of DNase hypersensitivity over early T cell development for known Notch target genes within the low-dose-responsive subset, including *Hes1* (Jarriault et al., 1995) and *Nrarp* (Pirot et al., 2004) as well as the aforementioned high-dose-dependent targets, are shown in Figures 3A and 3B, respectively.

## High-Dose-Dependent Gene Promoters Display Lower CpG Content than the Low-Dose-Responsive Subset

We next looked for a feature capable of distinguishing the promoters of Notch dose-responsive subgroups, focusing

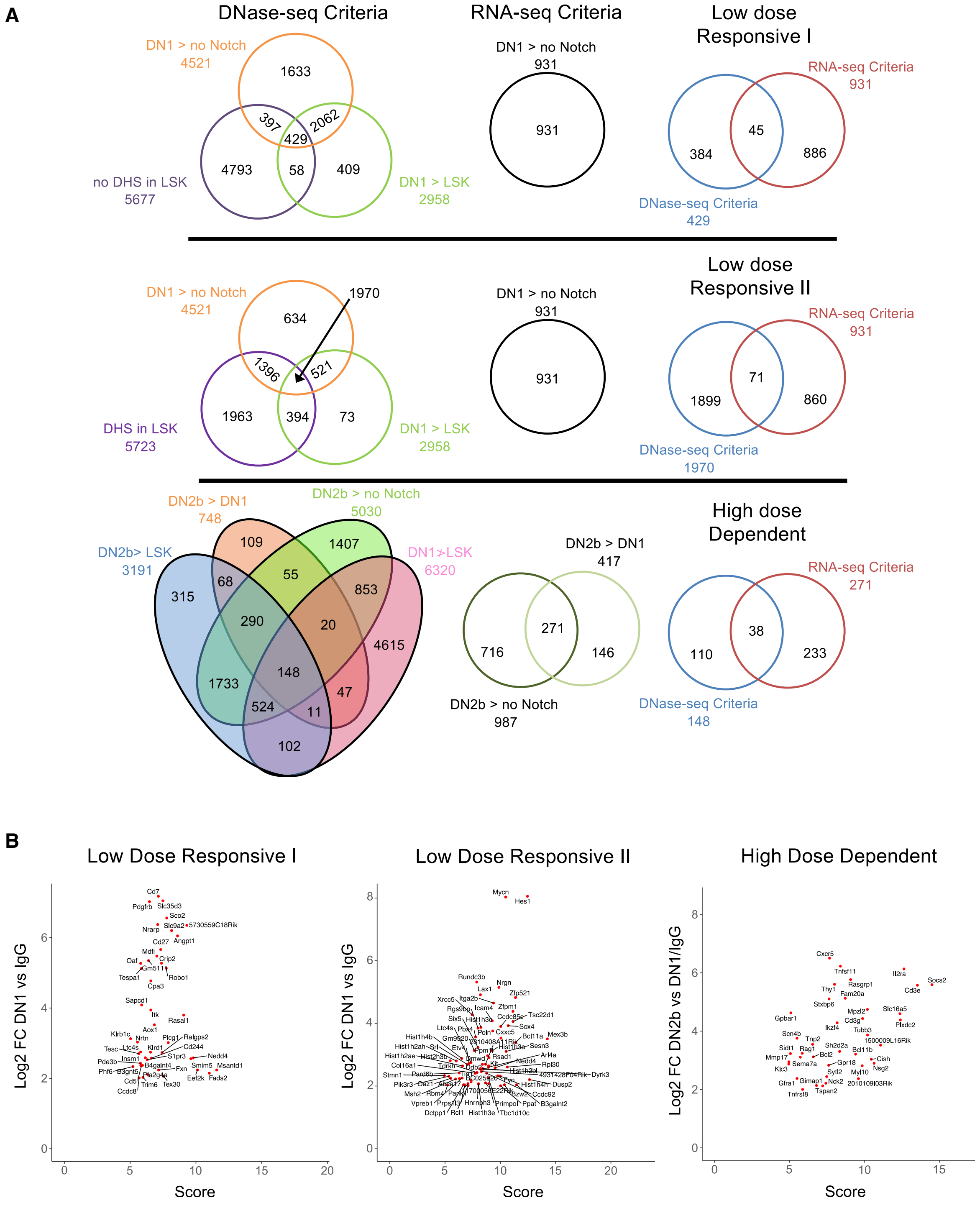


**Figure 2. Identification of Notch Dose-Responsive Gene Promoters**

(A) Venn diagrams representing the numbers and relationships between the categories of DNA-accessible regions defined as low-dose-responsive I, low-dose-responsive II, and high-dose-dependent.

(B) Scatterplots showing the correlation between DNA accessibility (x axis: score) and gene expression for genes (y axis: $\log_2$FC) under each criteria class.

See also Figures S2 and S3; Tables S1 and S2.

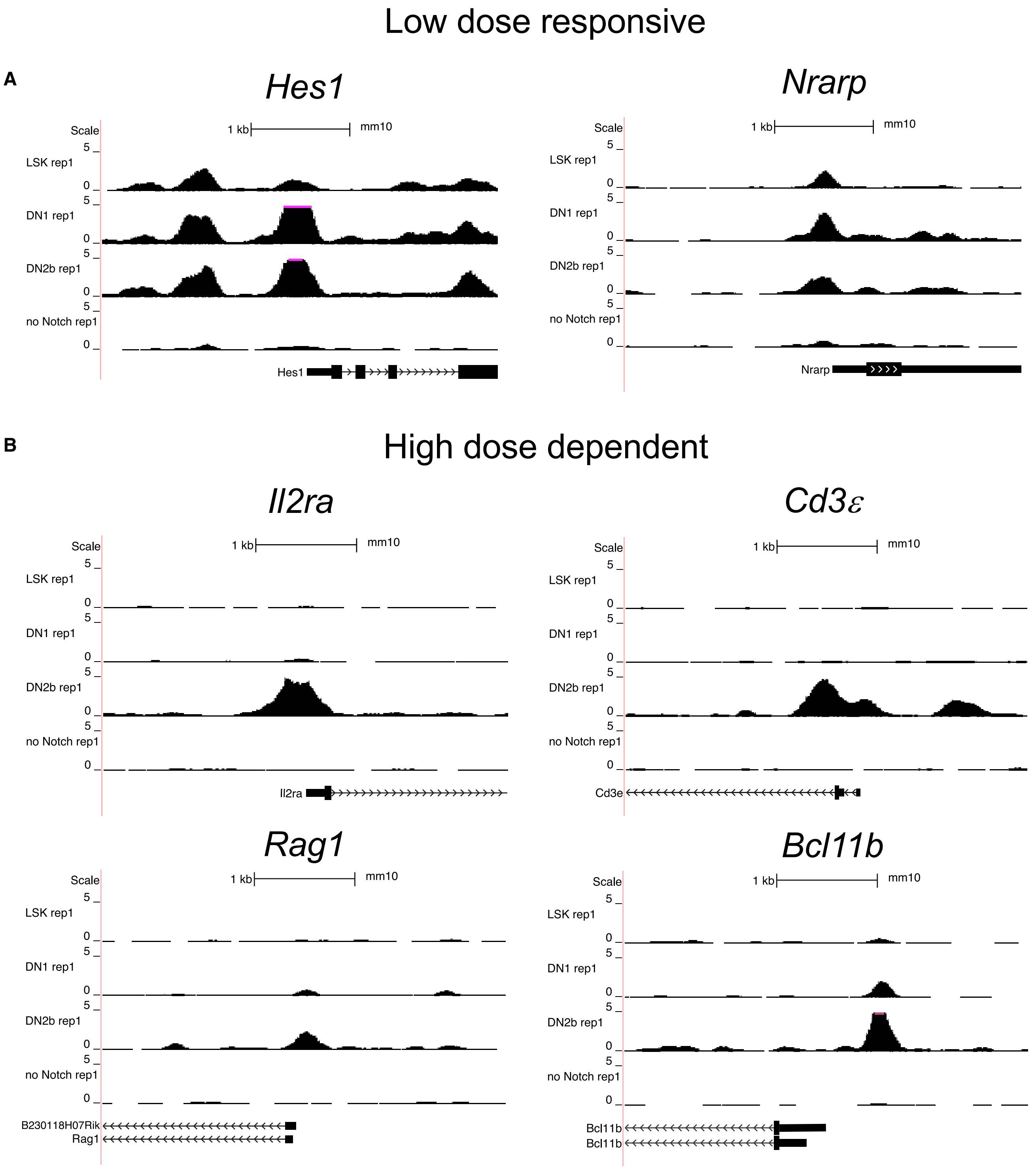


**Figure 3. DNase I Hypersensitivity at the Promoters of Key Notch Target Genes across Early T Cell Development**

LSK cells were isolated from murine marrow and cultured in the presence of high concentration of immobilized Delta1 ligand. Cells were harvested and flow sorted to isolate DN1 ($KIT^{++}CD44^{+}CD25^{-}$) and DN2b ($KIT^{+}CD44^{lo}CD25^{+}$) subpopulations. LSK cells were also grown in the absence of Notch signaling as a control. Nuclei were isolated from each cell population and treated with DNase I. Following DNase I digestion, small double-hit fragments were purified and sequencing libraries prepared. Paired-end sequencing was performed using the HiSeq platforms (Illumina). Normalized read density data from one replicate of each developmental stage was uploaded into the UCSC

*(legend continued on next page)*

on CpG content. LCG promoters are known to be enriched among lineage-associated genes (Barrera et al., 2008; Saxonov et al., 2006). Their lack of promoter histone methylation under non-differentiation conditions (Mikkelsen et al., 2007) as well as their high nucleosome occupancy (Teif et al., 2012) suggest that LCG promoters may keep high-dose-dependent Notch targets in a default inactive state. Therefore, we calculated the observed/expected CpG ratio among the promoters of Notch dose-responsive subgroups and found that the mean of observed/expected CpG ratio of high-dose-dependent promoters (0.427) was statistically lower (Mann-Whitney p value = $8.071 \times 10^{-5}$) than that of low-dose-responsive promoters (0.595) (Figure 4). In fact, three key high-dose-dependent targets essential for progression through T cell commitment display particularly low CpG content: *Il2ra* (0.14), *Cd3ε* (0.375), and *Rag1* (0.215) (Table S2). These observations suggest that LCG promoters enable Notch targets to act as gatekeepers of T cell development by requiring high levels of Notch signaling for gene activation, levels of signaling limited to the Notch ligand enriched thymus.

## DISCUSSION

Our study provides the first assessment of Notch dose-dependent changes in the DNA accessibility of target genes during T lineage development, identifying LCG promoters as a key regulator of Notch signaling threshold. Our findings suggest that promoter DNA inaccessibility at genes essential for T lineage commitment provides robust protection against stochastic activation in inappropriate Notch signaling contexts (Perdigoto et al., 2011), limiting T cell development to the thymus. Determining whether this paradigm regulates cell-fate outcome in other developmental contexts is of fundamental importance to understanding how Notch determines cell lineage.

Notch signaling is unique in that each ligand-activated receptor produces one Notch ICD capable of inducing transcription (Andersson et al., 2011). Without the means to amplify the transcriptional effector, the end result on gene expression is logically quantitative, a suggestion that has been validated both *in vitro* and *in vivo* (Dallas et al., 2005; Delaney et al., 2005; Gama-Norton et al., 2015). Recent *in vivo* studies have shown that cells respond to the amount of Notch ICD present by altering the burst duration of the transcriptional response (Falo-Sanjuan et al., 2019; Lee et al., 2019). *In vivo*, ligand identity has been shown to regulate Notch activation dynamics, whereby DLL1 induces pulses of NICD while DLL4 allows for more sustained NICD levels, signaling dynamics that impact target gene activation and, ultimately, cell-fate outcome (Nandagopal et al., 2018). As thymic DLL4 expression is required for NOTCH1-dependent T cell development (Hozumi et al., 2008), this suggests that *ex vivo* exposure of cells to high density of immobilized ligand mimics the sustained NICD levels generated following DLL4-mediated Notch activation.

While Notch is essential to elicit a change in DNA accessibility at the promoters of the aforementioned key T cell commitment genes, we cannot conclusively say that all of the high-dose-dependent genes identified are direct Notch targets. We recognize that the acquisition of DNA accessibility and gene expression at some high-Notch dose-dependent genes could be due to indirect effects of high Notch dosage, such that a handful of high-dose genes activate other genes classified as high dose dependent but are not necessarily bound by Notch. In fact, genes with LCG promoters have been shown to rely more heavily on transcription factors to activate their expression (Valen and Sandelin, 2011) and transcriptional coactivator usage has been shown to be a key regulator of Notch target gene selection throughout evolution (Bernard et al., 2010; Liu et al., 2010; Neves et al., 2007). Relevant to T cell development are well-documented cooperative interactions among transcription factors, including NOTCH, TCF1, and GATA3 (for a review see Yui and Rothenberg, 2014). Future perturbation studies will be required to determine the transcription factor network necessary to promote lineage-specific regulatory DNA accessibility in the context of quantitative differences in Notch signal strength.

## EXPERIMENTAL PROCEDURES

### Mice

C57BL/6J (Ly5.2) mice (Jackson Laboratory) were maintained and bred at the Fred Hutchinson Cancer Research Center. Mice bearing floxed *Notch1* were obtained from F. Radtke (EPFL, Switzerland) and maintained, bred, and deletion induced/verified using the methods described in Varnum-Finney et al. (2011).

### Cell Isolation and Culture

Using a FACSAria II cell sorter (BD), LSK cells were separated based on their high expression of SCA-1 and c-KIT from lineage-depleted bone marrow, as described in Dallas et al. (2005). Independent LSK isolates

genome browser. Each set of density profiles represents a 4-kb window for the labeled gene locus. The vertical axis represents tag density (in a 150-bp window) per million mapped reads. In black is a schematic of gene structure. The genomic scale of each locus has been noted using a 1kb scale bar.

(A) Mm10 coordinates for low-dose-responsive genes: ***Hes1*** chr16:30063271-30067271; ***Nrarp*** chr2:25178671-25182671.

(B) Mm10 coordinates for high-dose-dependent genes: ***Il2ra*** chr2:11640811-11644811; ***Cd3ε*** chr9:45007271-45011271; ***Bcl11b*** chr12:108002131-108006131; ***Rag1*** chr2: 101647611-101651611.

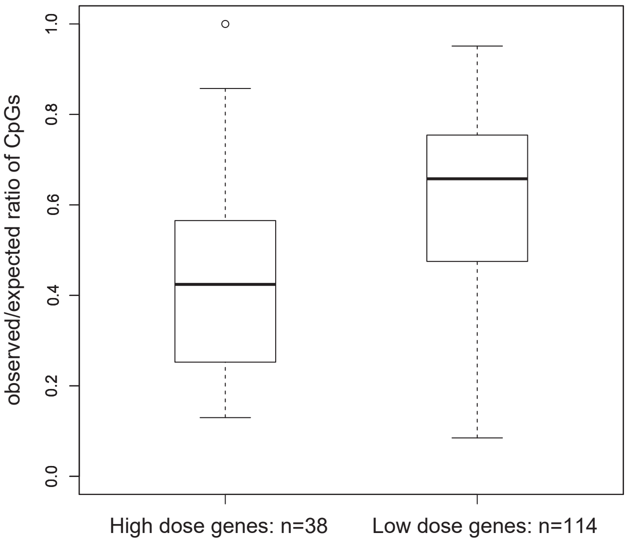


**Figure 4. High-Dose-Dependent Genes Display Lower Promoter CpG Content than the Low-Dose-Responsive Subset**
Box plot showing the observed/expected ratio of CpG content among high-dose-dependent and low-dose-responsive subsets. See also Table S2.

were cultured on immobilized Delta1 (Varnum-Finney et al., 2000), as previously described (Varnum-Finney et al., 2003). In brief, non-tissue culture-treated flasks (Nunc) were incubated overnight at 4°C with Delta1$^{ext\text{-}IgG}$ (0.75 or 5 μg/mL) or human $IgG_1$ (5 μg/mL, Sigma I4506) diluted in PBS, together with 5 μg/mL Retronectin (Takara). Flasks were washed extensively with PBS. Isolated LSK cells were added to prepared flasks in Iscove's modified Dulbecco's medium supplemented with 20% fetal bovine serum and 4GF (100 ng/mL murine SCF, human FLT-3 ligand, and human IL-6; 10 ng/mL human IL-11). Each Delta1$^{ext\text{-}IgG}$ culture was independently treated with IL-7 (100 ng/mL) at day 14 to drive cells further into T cell development. From each biological LSK replicate, DN1 ($KIT^{++}CD44^{+}CD25^{-}$) cells were isolated from 4GF conditions, whereas DN2a ($KIT^{++}CD44^{+}CD25^{+}$) and DN2b ($KIT^{+}CD44^{lo}CD25^{+}$) subpopulations were isolated from 4GF + IL-7 conditions using anti-mouse monoclonal antibodies CD25-APC-Cy7 (clone PC61, BD catalog no. 557658), CD44-APC (clone IM7, BD catalog no. 559250), and c-KIT-PE-Cy5 (clone 2B8, eBioscience catalog no. 15-1171-82).

## Dead Cell Removal from Cultured LSK Cells

Cells were incubated with microbeads (100 μL/$10^7$ cells; dead cell removal kit, Miltenyi) for 15 min at room temperature. Volume was increased with 1× binding buffer (supplied) to ensure $10^8$ cells or less per milliliter. Cells were then run through the Possel/Qrinse program on a Macs mini sampler (Miltenyi). Samples with >95% viability moved forward to nuclear isolation and DNase I treatment.

## Nuclear Isolation and DNase I Treatment

This protocol was adapted from John et al. (2013). Viable cells (5 × $10^6$) were spun at 1,400 rpm for 5 min at 4°C in a tabletop centrifuge and washed with 5 mL of cold PBS. Cells were resuspended in Lysis Buffer (10 mM Tris [pH 7.4], 10 mM NaCl, 3 mM $MgCl_2$, 150 μM spermine, and 500 μM spermidine, made in a 50-mL aliquot with one cOmplete EDTA-free protease inhibitor pellet [Roche]). An equal volume of 2× IGEPAL stock (0.2% IGEPAL diluted in Lysis Buffer) was added, mixed by inversion 10 times and incubated on ice for precisely 4 min. Nuclei were pelleted (5 min, 4°C, 1,750 rpm) and washed with 2 mL of Buffer A (100 mM NaCl, 50 mM Tris [pH 8.15], 3 mM $MgCl_2$, 150 μM spermine, and 500 μM spermidine) with 0.35 M sucrose. Nuclei were resuspended in 500 μL of Buffer A without sucrose + 1 mM $CaCl_2$, transferred to a LoBind Eppendorf tube with pre-aliquoted diluted DNase I (Sigma D4527-200KU), and incubated at 37°C for precisely 3 min. The reaction was terminated with 500 μL of stop buffer (100 mM NaCl, 50 mM Tris [pH 8.15], 0.1% SDS, and 100 mM EDTA [pH 8]) followed by the addition of 10 μL of Proteinase K (Sigma). Percent nuclear recovery was typically around 80%. A portion of the deproteinized, DNase I-treated and mock-treated samples were run on a 1% agarose gel, stained with SYBR Safe DNA gel stain (Life Technologies), and scanned with a Typhoon imager. The patterns observed were used to pick the appropriately digested samples for sequencing (John et al., 2013).

## DNase-seq and Data Analysis

Following DNase I digestion, purification of small double-hit fragments and sequencing library preparation was performed as in John et al. (2013). Paired-end sequencing was performed using the HiSeq 2000/2500 platforms (Illumina). DNase-seq datasets used in this study are available via the GEO (series accession GEO: GSE142739). Raw sequencing reads were trimmed to remove adapter sequences and aligned to the mouse genome (mm10, https://www.encodeproject.org/files/ENCFF340HIY/) using bwa (version 0.7.12) with the following parameters: "-Y -l 32 -n 0.04" and "-n 10 -a 750" for alignment and mate-pairing (aln and sampe, respectively). DHS peaks (i.e., hotspots) were determined using hotspot2 as outlined in https://www.encodeproject.org/pipelines/ENCPL202DNS/, and in total ~170,000 DHS regions were identified. A master list of peak regions was defined using the consensus DHS selection technique outlined in Thurman et al. (2012).

## Differential DNA Accessibility Analysis

DNase-seq data were collected from LSK cells and individual DN subpopulations (DN1, DN2a, DN2b) as well as LSK cells cultured in the absence of Notch (negative control: IgG), whereby each stage has 2–3 replicates derived from independent LSK isolates and, where needed, T cell inductions. Importantly, cells from each DN stage were harvested sequentially following T cell induction of biologically distinct LSK isolates. To find DNA-accessible regions genome wide, we used hotspot2 to call peaks at each developmental stage (https://www.encodeproject.org/pipelines/ENCPL202DNS/), and identified ~170,000 DHS regions (4 kb) in total. To identify Notch dose-dependent changes in DNA accessibility, we used the non-parametric method ChIPtest ((Wu et al., 2015): https://cran.r-project.org/web/packages/ChIPtest/index.html) to conduct pairwise comparisons on the same 4-kb DHS peak region between any of two stages (e.g., LSK versus DN1, DN1 versus DN2b) and assign a differential accessibility score. Two non-parametric scores determined DHS significance. TS kn is

defined by using kernel smoothing curves to identify the regions with differential DNA accessibility profiles (Wu et al., 2015). Ts Dnun, which is an extension of TS kn, is a more robust score that employs non-parametric statistics without smoothing, allowing for possible heteroscedasticity in error variance (unequal variance across samples). Significant differential DNA accessibility may present as a peak in one stage and no peak in the other stage (peak/no peak), as well as peak height differences or peak location shifts. To determine overlap of the center of each DHS peak with promoters, we utilized promoter annotation from Gencode vM12, focusing on protein-coding genes. We similarly assessed the fraction overlapping with enhancers using the catalog of candidate cCREs derived from the ENCODE Project Consortium (2020).

## RNA Isolation

Total RNA was extracted (Qiagen) from individual DN subpopulations (DN1, DN2a, DN2b) as well as LSK cells cultured in the absence of Notch (negative control: IgG), where each stage has 2–4 replicates derived from independent LSK isolates and, where needed, T cell inductions. RNA was quantified by Nanodrop and assessed for quality using either the Agilent Bioanalyzer or Tapestation. Samples with RNA integrity number values of >8 were submitted for library preparation and sequencing on a Hi-Seq2000 machine.

## Analysis of RNA-seq Data

RNA-seq datasets used in this study are available via the series accession GEO: GSE142739. Reads that failed to pass Illumina's base call quality threshold were removed. The first 36 bases from the first read end of each sample were aligned to mm10 using TopHat v2.12 (Kim et al., 2013). Bam files were sorted and indexed using samtools v0.1.19 (Li and Durbin, 2009). Counts for each gene were generated with htseq-count v0.6.1p1 using the "intersection-strict" overlapping mode (Anders et al., 2015). Genes with less than 1 count/million in at least two samples (equal to the number of samples in the smallest group) were removed. Starting number of genes with non-zero count sums across all samples = 21,271; genes post filter = 12,480.

## Gene Ontology Analysis

Lists of low-dose Notch-responsive and high-dose Notch-dependent genes were subjected to GO enrichment analysis using GOrilla (Eden et al., 2007, 2009). The background list included all genes displaying a Notch-dependent promoter DHS (see Table S2). Biological process was the ontology term assessed.

## CpG Content Analysis

FASTA files were created based on the combined low-dose-responsive or high-dose-dependent promoter coordinates. We calculated the observed/expected ratio of CpG using the following values: observed CpG is the number of occurrences of CpG within the 2-kb promoter sequence; expected CpG is calculated as (number of C × number of G)/length of sequence (Gardiner-Garden and Frommer, 1987).

## Statistics

For DNase-seq data analysis, DHS peaks (i.e., hotspots) were determined using hotspot2 by an FDR cutoff of 0.05, as outlined in https://www.encodeproject.org/pipelines/ENCPL202DNS/. Promoter-associated DHSs were screened for Notch responsiveness using ChIPtest (Wu, 2013; Wu et al., 2015). ChIPtest models the spatial profiles of read counts in each DHS region and uses a non-parametric test to develop a differential accessibility score. To control for multiple testing issues, we considered Bonferroni adjustment on the effective number of tests by combining p values from the overlapping regions (Lun and Smyth, 2016; Wu et al., 2015), and the threshold of DNA accessibility score is based on an adjusted p value of $<0.05$. For RNA-seq analysis, read counts for each DN stage or "no Notch" control samples were used in pairwise comparisons to calculate $\log_2$-FC values. Statistical significance in certain pairwise comparisons was defined as $\log_2 FC \geq 2$. For GO analysis, only GO terms with a p value of less than $10^{-3}$ corrected for multiple hypotheses are reported. For CpG content analysis, statistical significance in the observed/expected CpG ratio among the promoters of Notch dose-responsive subgroups was determined using a Mann-Whitney test.

## Study Approval

The murine studies presented herein were approved by the Institutional Animal Care and Use Committee at Fred Hutchinson Cancer Research Center, Seattle, WA.

## Data and Code Availability

DNase-seq and RNA-seq datasets used in this study are available via series accession GEO: GSE142739.

## SUPPLEMENTAL INFORMATION

Supplemental Information can be found online at https://doi.org/10.1016/j.stemcr.2021.02.017.

## AUTHOR CONTRIBUTIONS

S.F. and I.D.B. designed the experiments. S.F. and B.V.-F. performed the experiments. Q.W., R.S., W.M., J.A.S., S.F., and I.D.B. analyzed the data. S.F. and I.D.B. wrote the manuscript. All authors reviewed and approved the manuscript for publication.

## CONFLICTS OF INTEREST

The authors declare no competing interests.


## ACKNOWLEDGMENTS

The authors would like to thank Carolyn Brashem-Stein, David Flowers, Cynthia (Nourigat) McKay, Ryan Basom, Jeff Delrow, and Hamid Bolouri (Benaroya Research Institute) for technical assistance as well as Ellen Rothenberg (Caltech) and Warren Pear (UPenn) for their critical suggestions. This work was supported by grants from the National Institutes of Health: NHLBI U01 HL100395 and NIDDK R01 DK110563.

Received: July 10, 2020
Revised: February 19, 2021
Accepted: February 23, 2021
Published: March 25, 2021

## REFERENCES


Anders, S., and Huber, W. (2010). Differential expression analysis for sequence count data. Genome Biol. *11*, R106.

Anders, S., Pyl, P.T., and Huber, W. (2015). HTSeq—a Python framework to work with high-throughput sequencing data. Bioinformatics *31*, 166–169.

Andersson, E.R., Sandberg, R., and Lendahl, U. (2011). Notch signaling: simplicity in design, versatility in function. Development *138*, 3593–3612.

Artavanis-Tsakonas, S., Rand, M.D., and Lake, R.J. (1999). Notch signaling: cell fate control and signal integration in development. Science *284*, 770–776.

Barrera, L.O., Li, Z., Smith, A.D., Arden, K.C., Cavenee, W.K., Zhang, M.Q., Green, R.D., and Ren, B. (2008). Genome-wide mapping and analysis of active promoters in mouse embryonic stem cells and adult organs. Genome Res. *18*, 46–59.

Bernard, F., Krejci, A., Housden, B., Adryan, B., and Bray, S.J. (2010). Specificity of Notch pathway activation: twist controls the transcriptional output in adult muscle progenitors. Development *137*, 2633–2642.

Chen, E.L.Y., Thompson, P.K., and Zuniga-Pflucker, J.C. (2019). RBPJ-dependent Notch signaling initiates the T cell program in a subset of thymus-seeding progenitors. Nat. Immunol. *20*, 1456–1468.

Clevers, H., Lonberg, N., Dunlap, S., Lacy, E., and Terhorst, C. (1989). An enhancer located in a CpG-island 3′ to the TCR/CD3-epsilon gene confers T lymphocyte-specificity to its promoter. EMBO J. *8*, 2527–2535.

Dallas, M.H., Varnum-Finney, B., Delaney, C., Kato, K., and Bernstein, I.D. (2005). Density of the Notch ligand Delta1 determines generation of B and T cell precursors from hematopoietic stem cells. J. Exp. Med. *201*, 1361–1366.

De Smedt, M., Taghon, T., Van de Walle, I., De Smet, G., Leclercq, G., and Plum, J. (2007). Notch signaling induces cytoplasmic CD3 epsilon expression in human differentiating NK cells. Blood *110*, 2696–2703.

Delaney, C., Varnum-Finney, B., Aoyama, K., Brashem-Stein, C., and Bernstein, I.D. (2005). Dose-dependent effects of the Notch ligand Delta1 on ex vivo differentiation and in vivo marrow repopulating ability of cord blood cells. Blood *106*, 2693–2699.

Eden, E., Lipson, D., Yogev, S., and Yakhini, Z. (2007). Discovering motifs in ranked lists of DNA sequences. PLoS Comput. Biol. *3*, e39.

Eden, E., Navon, R., Steinfeld, I., Lipson, D., and Yakhini, Z. (2009). GOrilla: a tool for discovery and visualization of enriched GO terms in ranked gene lists. BMC Bioinformatics *10*, 48.

ENCODE Project Consortium, Moore, J.E., Purcaro, M.J., Pratt, H.E., Epstein, C.B., Shoresh, N., Adrian, J., Kawli, T., Davis, C.A., Dobin, A., et al. (2020). Expanded encyclopaedias of DNA elements in the human and mouse genomes. Nature *583*, 699–710.

Falo-Sanjuan, J., Lammers, N.C., Garcia, H.G., and Bray, S.J. (2019). Enhancer priming enables fast and sustained transcriptional responses to notch signaling. Dev. Cell *50*, 411–425.e18.

Gama-Norton, L., Ferrando, E., Ruiz-Herguido, C., Liu, Z., Guiu, J., Islam, A.B., Lee, S.U., Yan, M., Guidos, C.J., Lopez-Bigas, N., et al. (2015). Notch signal strength controls cell fate in the haemogenic endothelium. Nat. Commun. *6*, 8510.

Gardiner-Garden, M., and Frommer, M. (1987). CpG islands in vertebrate genomes. J. Mol. Biol. *196*, 261–282.

Hozumi, K., Mailhos, C., Negishi, N., Hirano, K., Yahata, T., Ando, K., Zuklys, S., Hollander, G.A., Shima, D.T., and Habu, S. (2008). Delta-like 4 is indispensable in thymic environment specific for T cell development. J. Exp. Med. *205*, 2507–2513.

Jarriault, S., Brou, C., Logeat, F., Schroeter, E.H., Kopan, R., and Israel, A. (1995). Signalling downstream of activated mammalian Notch. Nature *377*, 355–358.

John, S., Sabo, P.J., Canfield, T.K., Lee, K., Vong, S., Weaver, M., Wang, H., Vierstra, J., Reynolds, A.P., Thurman, R.E., et al. (2013). Genome-scale mapping of DNase I hypersensitivity. Curr. Protoc. Mol. Biol. *Chapter 27*, Unit 21 27.

Kim, D., Pertea, G., Trapnell, C., Pimentel, H., Kelley, R., and Salzberg, S.L. (2013). TopHat2: accurate alignment of transcriptomes in the presence of insertions, deletions and gene fusions. Genome Biol. *14*, R36.

Kopan, R., and Ilagan, M.X. (2009). The canonical Notch signaling pathway: unfolding the activation mechanism. Cell *137*, 216–233.

Lai, E.C. (2004). Notch signaling: control of cell communication and cell fate. Development *131*, 965–973.

Lee, C., Shin, H., and Kimble, J. (2019). Dynamics of notch-dependent transcriptional bursting in its native context. Dev. Cell *50*, 426–435.e24.

Li, H., and Durbin, R. (2009). Fast and accurate short read alignment with Burrows-Wheeler transform. Bioinformatics *25*, 1754–1760.

Li, L., Leid, M., and Rothenberg, E.V. (2010). An early T cell lineage commitment checkpoint dependent on the transcription factor Bcl11b. Science *329*, 89–93.

Liu, H., Chi, A.W., Arnett, K.L., Chiang, M.Y., Xu, L., Shestova, O., Wang, H., Li, Y.M., Bhandoola, A., Aster, J.C., et al. (2010). Notch dimerization is required for leukemogenesis and T-cell development. Genes Dev. *24*, 2395–2407.

Liu, Z., Brunskill, E., Varnum-Finney, B., Zhang, C., Zhang, A., Jay, P.Y., Bernstein, I., Morimoto, M., and Kopan, R. (2015). The intracellular domains of Notch1 and Notch2 are functionally equivalent during development and carcinogenesis. Development *142*, 2452–2463.

Lun, A.T., and Smyth, G.K. (2016). csaw: a Bioconductor package for differential binding analysis of ChIP-seq data using sliding windows. Nucleic Acids Res. *44*, e45.

Maillard, I., Tu, L., Sambandam, A., Yashiro-Ohtani, Y., Millholland, J., Keeshan, K., Shestova, O., Xu, L., Bhandoola, A., and Pear, W.S. (2006). The requirement for Notch signaling at the beta-selection checkpoint in vivo is absolute and independent of the pre-T cell receptor. J. Exp. Med. *203*, 2239–2245.

McBlane, J.F., van Gent, D.C., Ramsden, D.A., Romeo, C., Cuomo, C.A., Gellert, M., and Oettinger, M.A. (1995). Cleavage at a V(D)J recombination signal requires only RAG1 and RAG2 proteins and occurs in two steps. Cell *83*, 387–395.

Mikkelsen, T.S., Ku, M., Jaffe, D.B., Issac, B., Lieberman, E., Giannoukos, G., Alvarez, P., Brockman, W., Kim, T.K., Koche, R.P., et al. (2007). Genome-wide maps of chromatin state in pluripotent and lineage-committed cells. Nature *448*, 553–560.

Nandagopal, N., Santat, L.A., LeBon, L., Sprinzak, D., Bronner, M.E., and Elowitz, M.B. (2018). Dynamic ligand discrimination in the notch signaling pathway. Cell *172*, 869–880.e19.

Neves, A., English, K., and Priess, J.R. (2007). Notch-GATA synergy promotes endoderm-specific expression of ref-1 in C. elegans. Development *134*, 4459–4468.

Perdigoto, C.N., Schweisguth, F., and Bardin, A.J. (2011). Distinct levels of Notch activity for commitment and terminal differentiation of stem cells in the adult fly intestine. Development *138*, 4585–4595.

Pirot, P., van Grunsven, L.A., Marine, J.C., Huylebroeck, D., and Bellefroid, E.J. (2004). Direct regulation of the Nrarp gene promoter by the Notch signaling pathway. Biochem. Biophys. Res. Commun. *322*, 526–534.

Radtke, F., Wilson, A., Stark, G., Bauer, M., van Meerwijk, J., MacDonald, H.R., and Aguet, M. (1999). Deficient T cell fate specification in mice with an induced inactivation of Notch1. Immunity *10*, 547–558.

Saxonov, S., Berg, P., and Brutlag, D.L. (2006). A genome-wide analysis of CpG dinucleotides in the human genome distinguishes two distinct classes of promoters. Proc. Natl. Acad. Sci. U S A *103*, 1412–1417.

Stalder, J., Larsen, A., Engel, J.D., Dolan, M., Groudine, M., and Weintraub, H. (1980). Tissue-specific DNA cleavages in the globin chromatin domain introduced by DNAase I. Cell *20*, 451–460.

Stergachis, A.B., Neph, S., Reynolds, A., Humbert, R., Miller, B., Paige, S.L., Vernot, B., Cheng, J.B., Thurman, R.E., Sandstrom, R., et al. (2013). Developmental fate and cellular maturity encoded in human regulatory DNA landscapes. Cell *154*, 888–903.

Teif, V.B., Vainshtein, Y., Caudron-Herger, M., Mallm, J.P., Marth, C., Hofer, T., and Rippe, K. (2012). Genome-wide nucleosome positioning during embryonic stem cell development. Nat. Struct. Mol. Biol. *19*, 1185–1192.

Thurman, R.E., Rynes, E., Humbert, R., Vierstra, J., Maurano, M.T., Haugen, E., Sheffield, N.C., Stergachis, A.B., Wang, H., Vernot, B., et al. (2012). The accessible chromatin landscape of the human genome. Nature *489*, 75–82.

Valen, E., and Sandelin, A. (2011). Genomic and chromatin signals underlying transcription start-site selection. Trends Genet. *27*, 475–485.

Varnum-Finney, B., Wu, L., Yu, M., Brashem-Stein, C., Staats, S., Flowers, D., Griffin, J.D., and Bernstein, I.D. (2000). Immobilization of Notch ligand, Delta-1, is required for induction of notch signaling. J. Cell Sci. *113* (Pt 23), 4313–4318.

Varnum-Finney, B., Brashem-Stein, C., and Bernstein, I.D. (2003). Combined effects of Notch signaling and cytokines induce a multiple log increase in precursors with lymphoid and myeloid reconstituting ability. Blood *101*, 1784–1789.

Varnum-Finney, B., Halasz, L.M., Sun, M., Gridley, T., Radtke, F., and Bernstein, I.D. (2011). Notch2 governs the rate of generation of mouse long- and short-term repopulating stem cells. J. Clin. Invest. *121*, 1207–1216.

Vierstra, J., Rynes, E., Sandstrom, R., Zhang, M., Canfield, T., Hansen, R.S., Stehling-Sun, S., Sabo, P.J., Byron, R., Humbert, R., et al. (2014). Mouse regulatory DNA landscapes reveal global principles of cis-regulatory evolution. Science *346*, 1007–1012.

Washburn, T., Schweighoffer, E., Gridley, T., Chang, D., Fowlkes, B.J., Cado, D., and Robey, E. (1997). Notch activity influences the alphabeta versus gammadelta T cell lineage decision. Cell *88*, 833–843.

Wu, Q. (2013). Statistical Methods for Analysis of Multi-Sample Copy Number Variants and ChIP-seq Data (University of Pennsylvania).

Wu, C., Bingham, P.M., Livak, K.J., Holmgren, R., and Elgin, S.C. (1979a). The chromatin structure of specific genes: I. Evidence for higher order domains of defined DNA sequence. Cell *16*, 797–806.

Wu, C., Wong, Y.C., and Elgin, S.C. (1979b). The chromatin structure of specific genes: II. Disruption of chromatin structure during gene activity. Cell *16*, 807–814.

Wu, Q., Won, K.J., and Li, H. (2015). Nonparametric tests for differential histone enrichment with ChIP-seq data. Cancer Inform. *14*, 11–22.

Yui, M.A., and Rothenberg, E.V. (2014). Developmental gene networks: a triathlon on the course to T cell identity. Nat. Rev. Immunol. *14*, 529–545.

Zhang, Y., Liu, T., Meyer, C.A., Eeckhoute, J., Johnson, D.S., Bernstein, B.E., Nusbaum, C., Myers, R.M., Brown, M., Li, W., et al. (2008). Model-based analysis of ChIP-Seq (MACS). Genome Biol. *9*, R137.

Zuniga-Pflucker, J.C. (2004). T-cell development made simple. Nat. Rev. Immunol. *4*, 67–72.

Stem Cell Reports, Volume *16*

## Supplemental Information

# Inaccessible LCG Promoters Act as Safeguards to Restrict T Cell Development to Appropriate Notch Signaling Environments

**Suzanne Furuyama, Qian "Vicky" Wu, Barbara Varnum-Finney, Richard Sandstrom, Wouter Meuleman, John A. Stamatoyannopoulos, and Irwin D. Bernstein**

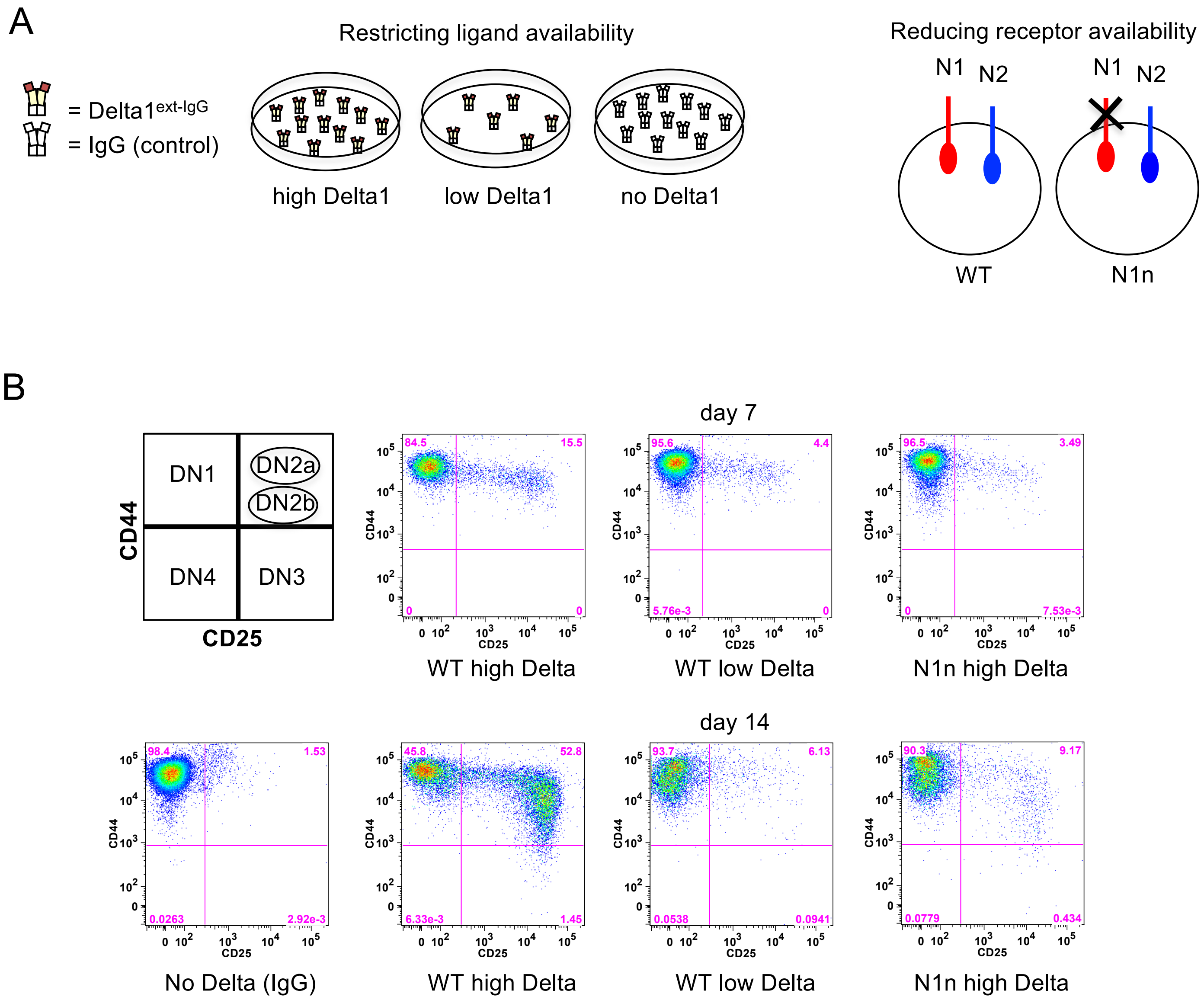


**Figure S1: Use of an immobilized Notch ligand culture system to control the level of Notch activation, and ultimately cell fate outcome, Related to Figure 1. A.** Schematic representation of the ex vivo culture system used. To vary the amount of ligand presented during ex vivo culture, flasks are coated with low (0.75μg/ml) or high (5μg/ml) concentration of Delta1$^{ext\text{-}IgG}$. To reduce the amount of available Notch receptor, we utilized a mouse strain bearing a Cre-mediated conditional *Notch1* deletion (Radtke et al., 1999). **B.** Representative dot plots showing the percent of CD25$^{+}$CD44$^{+}$ Double Negative 2 (DN2) precursors generated following 7 and 14 days of LSK culture on different doses of Delta1$^{ext\text{-}IgG}$ or no Delta (IgG) control. Numbers within dot plots denote the percentage of the population within the respective quadrants.

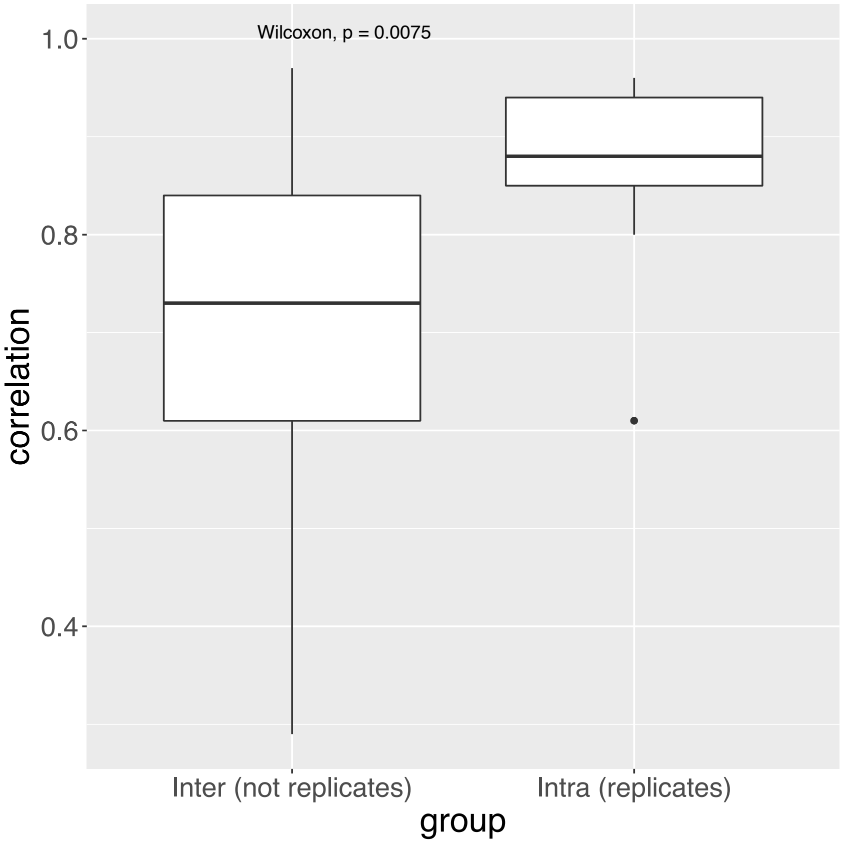


**Figure S2: Assessment of DNase-seq replicate concordance, Related to Figures 1 & 2.** Boxplot describing the correlation between datasets from the same developmental stage (Intra) as well as among different developmental stages (Inter). The median correlation coefficient, R, among intra-stage replicates is 0.88, while that of inter-stage non-replicates is 0.73. The non-parametric Wilcoxon rank test was used to assess statistical significance among groups, p = 0.0075.

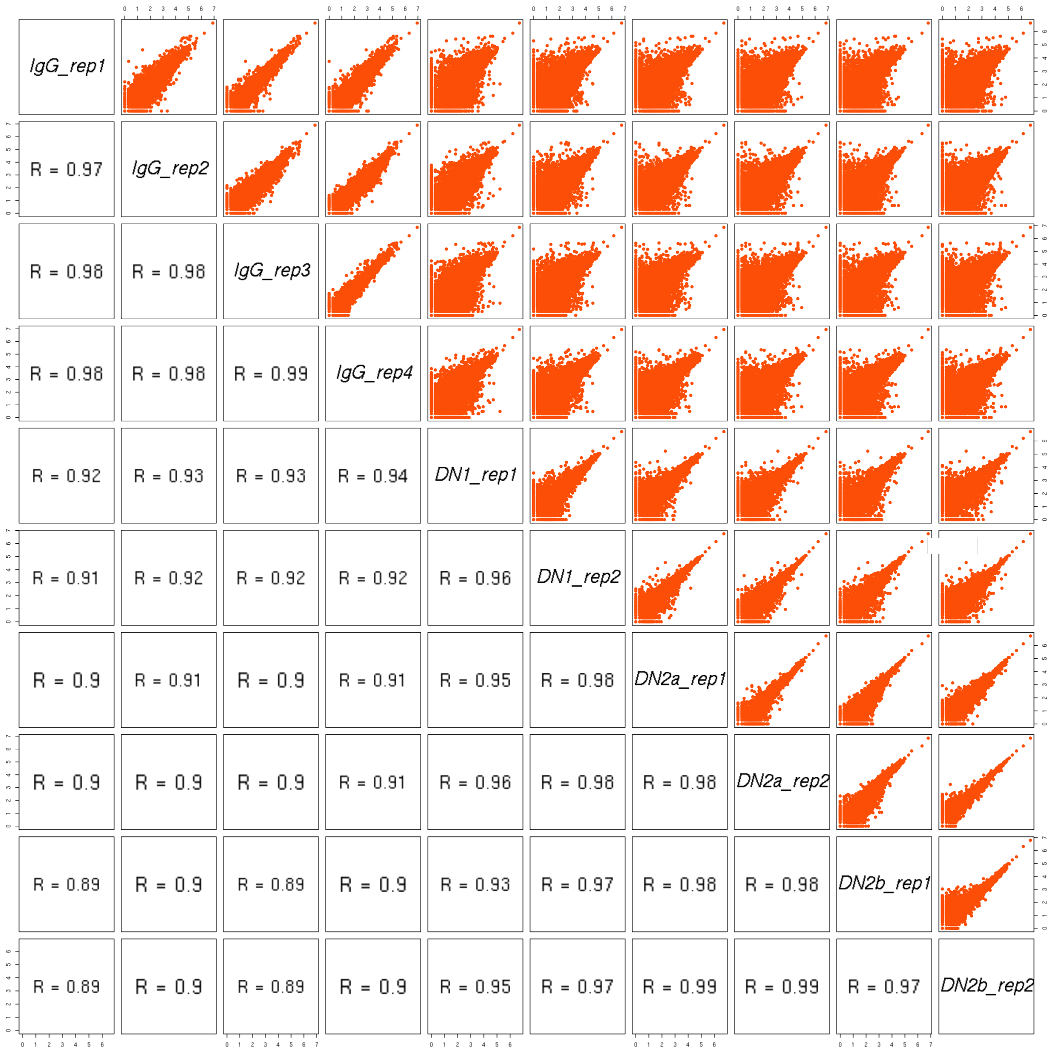


**Figure S3: Assessment of RNA-seq replicate concordance, Related to Figure 2.** Scatterplots showing the correlation between each dataset, both the same (biological replicates) and different (non-biological replicates) developmental stages. The X and Y-axes represent $\log_{10}$(gene expression count +1). The correlation coefficient, R, is also noted.

**Supplemental Table Legends:**

**Table S1: DNase-seq & RNA-seq Dosage Screen Results, Related to Figure 2.**

Sheet 1: ***Master Table***. Includes all DNase-seq and RNA-seq results used to identify high dose Notch dependent and low dose Notch responsive promoters.
Sheets 2-4: '***Unique***' sheets represent a simplified list of unique gene promoters and their coordinates that overlap with a DHS in each dosage class (high dose, low dose part1, low dose part2).

**Table S2: Gene Ontology and CpG analysis, Related to Figures 2 & 4.**

Sheet 1: Reference gene list for Gene Ontology Analysis (GOrilla).
Sheet 2: GOrilla output for combined low dose Notch responsive genes.
Sheet 3: GOrilla output for high dose Notch dependent genes.
Sheet 4**:** Observed/expected CpG ratio among the promoters of Notch dose responsive subgroups
